# EEG Theta/Beta Ratio Neurofeedback Training in Healthy Females

**DOI:** 10.1007/s10484-020-09472-1

**Published:** 2020-05-26

**Authors:** Dana van Son, Willem van der Does, Guido P. H. Band, Peter Putman

**Affiliations:** 1 Institute of Psychology, Leiden University, Leiden, The Netherlands; 2 Leiden Institute for Brain and Cognition, Leiden, The Netherlands

**Keywords:** EEG theta/beta ratio, Neurofeedback, Multiple baseline design

## Abstract

A growing number of studies suggest that EEG theta/beta ratio (TBR) is inversely related to executive cognitive control. Neurofeedback training aimed at reducing TBR (TBR NFT) might provide a tool to study causality in this relation and might enhance human performance. To investigate whether TBR NFT reduces TBR in healthy participants. Twelve healthy female participants were assigned (single blind) to one of three groups. Groups differed on baseline durations and one group received only sham NFT. TBR NFT consisted of eight or fourteen 25-min sessions. No evidence was found that TBR NFT had any effect on TBR. The current TBR NFT protocol is possibly ineffective. This is in line with a previous study with a different protocol.

## Introduction

Resting state encephalographic (EEG) signals are composed of different frequency components, many of which are found to be relatively stable over time (Williams et al. 2005). Specific spectrum components reflect functional neural activity as an electrophysiological correlate with certain behaviors (Hofman and Schutter 2011; Sutton and Davidson 2000). For example, the ratio between activity in the theta band (4–7 Hz) and activity in the beta band (13–30 Hz), the theta/beta ratio (TBR), has been related to different aspects of cognitive control and motivated decision making (Massar et al. 2014; Massar et al. 2012; Schutter and van Honk 2005), to attentional control in healthy young adults (Angelidis et al. 2016; Putman et al. 2010, 2014; van Son et al. 2018), to off-task thoughts (i.e. mind-wandering; van Son et al. 2019a, b) and to reversal learning (Wischnewski et al. 2016). Additionally, a higher baseline TBR was found to correlate to a stronger decline in cognitive control after stress-induction (Putman et al. 2014). TBR has a high test–retest reliability and predicts attentional control scores over a one-week interval (Angelidis et al. 2016). All in all, TBR is likely a stable electrophysiological marker of executive control.

Attentional control is the ability to strategically deploy top-down controlled attention over bottom-up information processing (for instance intrusive anxious cognitions; see Verwoerd et al. 2008) to support performance of goal-directed tasks (Derryberry and Reed 2002). Lower levels of attentional control have, amongst others, been associated with general anxiety disorder (GAD; Amir et al. 2009). In GAD, anxiogenic cognitions take the form of perseverative worry, which consists of repetitive thoughts about everyday concerns (Armstrong et al. 2011; Burns et al. 1996) and are thought to start as uninhibited selective bottom-up thought-processing, akin to automatic attentional processing of threat-information (Hirsch and Mathews 2012). Along these lines, reduced attentional control has also been found related to stronger attentional bias towards threat (e.g. Bardeen and Orcutt 2011; Derryberry and Reed 2002; Mogg and Bradley 2016). Attentional bias occurs when stress or anxiety prioritize the processing of mildly threatening distracters; during anxious states, bottom-up processing of threatening distracters is facilitated, while top-down executive functions are inhibited (Derakshan and Eysenck 2009). Furthermore, executive control can be decreased by the anxious distracting thoughts impairing working memory (Coy et al. 2011; Eysenck et al. 2007). This is in line with the widely accepted idea that test anxiety causes divided attention, leading to for example lower academic performance (Hembree 1988; Duty et al. 2016).

TBR was found to be related to trait attentional control (Angelidis et al. 2016; Putman et al. 2010, 2014; van Son et al. 2018), to resilience to the effects of stress on task performance (Putman et al. 2014), to down-regulation of negative affect (Tortella-Feliu et al. 2014) and to regulation of automatic attentional bias to threat; (Angelidis et al. 2018; van Son et al. 2018). The study of TBR is therefore potentially interesting for a range of phenomena, conditions and applications, such as stress-cognition interactions, anxious psychopathology or human performance enhancement. Experimentally manipulating TBR could give further insights in causal relations between this EEG marker, cognitive control and stress effects, as well as possibly pave the way for future development of interventions.

A method to induce changes in TBR is neurofeedback training (NFT). NFT is a procedure in which participants may implicitly learn to gain control over particular aspects of their EEG signal. Providing online feedback on people’s EEG spectrum while asking them indirectly to increase or decrease power in certain frequency bands (e.g. by keeping a video running) can eventually lead to the ability to do this (Vernon 2005). An increasing number of studies have reported positive effects of NFT in neurological and psychological disorders (Marzbani et al. 2016) as well as areas like performance enhancement (for a review, see Gruzelier 2014a) optimized performance in sports (Graczyk et al. 2014), cognitive control (Keizer et al. 2010), improved self-regulation skills and motor system excitability (Studer et al. 2014) and situations with counterproductive interactions between stress and cognition, such as music performance under stressful conditions (Egner and Gruzelier 2003). NFT has also been applied for reducing symptoms of Attention Deficit Hyperactivity Disorder (ADHD). ADHD has often been associated with high TBR (for review and meta-analysis, see Arns et al. 2013; Barry et al. 2003) and NFT targeting TBR has been found to successfully reduce TBR and ADHD-related symptoms in individuals diagnosed with ADHD (e.g. hyperactivity, impaired attention; e.g. Butnik 2005; Leins et al. 2007; Lubar et al. 1995; Kouijzer et al. 2009), though some studies report absence of long-term effects (e.g., (Janssen et al. 2020).

The study of the potential beneficial effects of TBR-reducing NFT seems warranted given the abovementioned relations between TBR and various psychological regulatory constructs. However, because we believe it is imperative to first ascertain that indeed reliable changes in TBR by NFT can be observed, we selected healthy participants with mildly elevated TBR. We aimed to investigate whether NFT induces changes in TBR in people with mildly elevated TBR but who do not have a clinical diagnosis of psychopathology. The primary outcome measure of this study was changes in the targeted EEG parameters. These changes are likely easier to detect and more consistent than changes at the more multifaceted and complex behavioral level.

Doppelmayr and Weber (2011) previously investigated whether a TBR NFT protocol exerts the intended effects on the EEG spectrum level in healthy individuals, not selected on TBR level. The effect of the TBR NFT training on its trained EEG indices was compared to the effect of an NFT protocol training the ‘Sensori-Motor Rhythms’ (SMR; 12–15 Hz) and a sham-NFT with daily changing frequency bands. Healthy individuals who received the SMR training protocol were able to significantly modulate their EEG in the trained frequency band, whereas individuals who received the TBR or sham protocol were not. To our knowledge, this is the only study to date that directly investigated TBR NFT in healthy individuals by primarily looking at the direct effects on the EEG theta and beta parameters. We aimed to replicate and extend Doppelmayr & Weber’s findings by testing in an independent study again if TBR can be changed.

Our hypothesis that a TBR NFT can induce changes in EEG for individuals with mildly elevated TBR has not been studied extensively yet. Subjecting participants from this population to a very lengthy active TBR NFT training is demanding on the participants and could potentially cause unknown side effects. The best approach would be to study a small sample in depth, by thoroughly inspecting effects of active-NFT in each individual per session. We therefore employed a multiple baseline case series design. This design was chosen in order to closely examine any possible change in TBR at the level of the studied individuals so as not to overlook possible leads to increase NFT effectiveness and to minimize the chance of prematurely ruling out potential effectiveness of NFT for our purposes. A multiple baseline case series design involves the measurement of multiple persons both before and after an intervention (Watson and Workman 1981). In this design the start of the intervention is varied sequentially across individuals or small groups of individuals. During the baseline phase before intervention, the behavior or measure of interest is measured a number of times to observe its natural variation over time. When a change only takes place shortly after a specific intervention is introduced and not following a different intervention, the change can be attributed to the intervention (Baer et al. 1968; Kinugasa et al. 2004; Koehler and Levin 2000). A frequently used method in a case series design is visual inspection, which provides a reliable alternative for statistical tests for detecting changes by intervention, when sample sizes are too small for good statistical power (Fisher et al. 2003).

There are other, mainly ethical benefits to the smaller number of sessions required for a case series design. First of all, executing a controlled study with large sample sizes (see Cohen et al. 2013) places a lot of burden on the test-participants and implies a big investment of societal resources that may not be warranted yet. Additionally, nothing is known about possible negative side-effects in our intended population although the literature does suggest that such effects might exist. Low TBR for example has been related to low approach-driven or hedonically motivated behavior as measured with the IOWA gambling task (which has been associated with depression and anxiety; Cella et al. 2010; Massar et al. 2012; Massar et al. 2014; Mueller et al. 2010; Schutter and van Honk 2005) and as measured with the self-report BIS/BAS scale (Putman et al. 2010; Carver and White 1994). Also, two studies (Putman et al. 2010; Angelidis et al. 2016) demonstrate a negative association between TBR and self-reported negative, anxious affect as measured with the State-Trait Anxiety Inventory (Spielberger et al. 1983; van der Ploeg and Defares 1980). Finally, one study (Enriquez-Geppert et al. 2014) has reported beneficial effects of working memory performance of a theta-only upregulation using NFT in healthy participants. All in all, at this stage of TBR NFT research in healthy adults, where side effects are not yet thoroughly investigated but cannot be ruled out, applying this intervention in a large group of participants and over a long time period is not yet defensible.

**Fig. 1:**
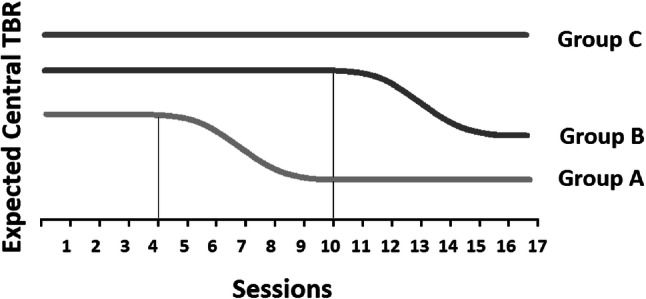
Expected pattern of central TBR per group. Central TBR in Group A and B was expected to reduce some time after onset of active-NFT (session 4 for Group A and session 10 for Group B). The reduction is expected to be relatively constant between individuals. Central TBR in Group C was not expected to show any change over time because sham-NFT was introduced in session 4 instead of active-NFT. The thin black line represents the point of active-NFT introduction for Group A and Group B

We assessed whether NFT reduces TBR in healthy individuals with mildly elevated TBR. TBR was the primary outcome; self-reported attentional control and state anxiety were assessed as secondary outcomes and to measure potentially unwanted side effects of NFT. A multiple baseline design was used employing various durations of baseline, sham-NFT and active-NFT sessions. We expected to see a reduction of TBR sometime after switching from measurement-only sessions to active treatment. We expected an absence of such measurement-only-controlled changes in a third sham-only group and, finally, we expected that TBR during the final sessions would be clearly lower in the two active NFT groups than in the sham-only group (see Fig. 1). Our primary interest was changes in TBR within the training sessions but we also looked at changes in TBR during resting state measurements at the start of the sessions (between-sessions changes). Finally, we performed in-depth exploration of the time course of TBR within and between training sessions, exploiting the case series’ benefits of temporally fine-grained observation.

## Methods

### Participants

Twelve female participants (age 19–23 years; *M* = 21; *SD* = 1.04) were included by preselection on elevated resting state frontal TBR from three previous studies from our lab (in which no attempts to change EEG measures were made in any way). Because of the low number of men in these previous studies, only female participants were included in the current study. The preselection was done based on frontal TBR measures obtained from previous studies in our lab in unselected female participants who left contact details for further study (*N* = 54). Frontal TBR was chosen as preselection outcome variable since all previous studies had frontal TBR as main outcome variable and this is highly correlated with central TBR (*r* = 0.902, *p* < 0.001 in the current study). We invited participants with the highest frontal TBR for the current study. Other inclusion criteria were; age between 18 and 24 years old; no history of neurologic or psychiatric disorders; no history or current use of recreational drugs that can affect the central nervous system (CNS) other than low to moderate alcohol use or nicotine use and no use of medication that is known to directly influence the CNS. Recruitment took place at Leiden University, The Netherlands, between December 2015 and February 2016. All participants signed informed consent and were free to terminate their participation at any time. For monetary compensation we used an incremental pay-off scheme, including disproportionately larger rewards for longer participation. This pay-off scheme was applied to minimize drop-out from the study. The study was approved by the local ethics review board (CEP16-011,413), and pre-registered at ClinicalTrials.gov (NCT02763618).

### Design

**Fig. 2:**
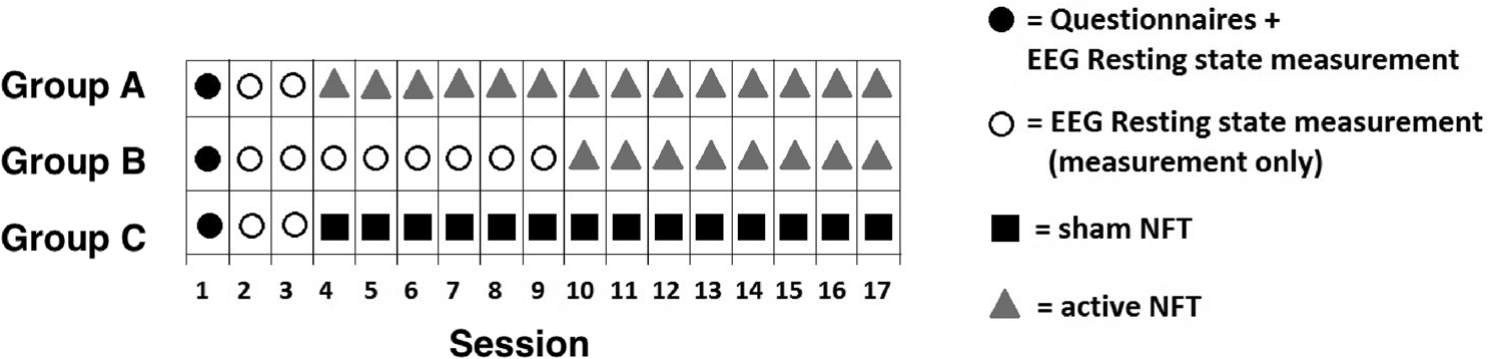
Difference in session course per group

A single-blind case series multiple baseline design was used with a baseline (measurement-only period) varying prior to training onset and after training offset (Fig. 2). Before the first lab session, the participants were assigned to one of three groups. Care was taken to obtain a more or less equal distribution of frontal TBR levels and age across the groups, but other than that the allocation to one of the three study groups was arbitrary. Allocation was done by the principal investigator who was not involved in the actual testing of the participants and had no contact with them. All participants started with a three-session measurement-only phase with only a resting state EEG measurement. Participants in Group A continued with 14 sessions of active-NFT. Group B received six extra measurement-only sessions before they continued with eight active-NFT sessions. Group C received 14 sham-NFT sessions after the three-session measurement-only phase. A minimum of eight sessions active-NFT was applied because changes were usually seen around five or six 30-min sessions in studies that found effects on theta frequency (Kao et al. 2014; Enriquez-Geppert et al. 2014). All participants were blind to which group they were in but the experimenters were not blind to this for reasons of practical feasibility. During all sessions, questionnaires for state anxiety and state attentional control were assessed before every EEG measurement, active-NFT or sham-NFT. Our primary outcome variable was changes in frontal TBR within each session while our secondary outcome measurement was changes in frontal TBR between the sessions.

### Materials

#### Self-Report Questionnaires

During the first and last session, participants completed the trait version of the State-Trait Anxiety Inventory (STAI-T; Spielberger et al. 1983; van der Ploeg and Defares 1980) and the Attentional Control Scale (ACS; Derryberry and Reed 2002). The STAI-T assesses trait anxiety (20 items, range 20–80; Cronbach’s alpha in current study = 0.89) and participants had to indicate their agreement with items like ‘I feel satisfied with myself’ and ‘I am a steady person’ on a four-point Likert scale. The ACS assesses self-reported attentional control in terms of attentional inhibition, attentional focus and the capacity to generate new thoughts (20 items, range 20–80; Cronbach’s alpha in current study = 0.85), e.g. ‘I can quickly switch from one task to another’. Self-reported state anxiety and state attentional control were measured on every session using the state version of the State-Trait Anxiety Inventory (STAI-S; Spielberger et al. 1983; van der Ploeg and Defares 1980) and the State-Attentional Control Scale (SACS; Angelidis et al. 2019). STAI-S measures state anxiety at the moment of participation (20 items, range 20–80, Cronbach’s alpha in current study = 0.91) and includes items like ‘I am tense’. SACS measures attentional control at the moment of participation (six items) and included items like ‘I feel very focused’ (Cronbach’s alpha in current study = 0.84). The Behavioral Activation Scale (BAS; part of the Behavioral Inhibition and Activation Scale; BIS/BAS Carver and White 1994) was assessed for the personality trait of behavioral activation (Cronbach’s alpha in current study = 0.78). The BAS consists of the subscales BAS Reward, BAS Drive and BAS Fun Seeking but we assessed the total BAS score. STAI-T, ACS and BAS were included only to see if their scores changed on these measures on the first session compared to the last session to check for potential unwanted side-effects. STAI-S and SACS were used to observe possible unwanted side-effects of NFT over time with a greater precision. The questionnaires were programmed and presented using E-Prime 2.0 software (Psychology Software Tools, Pittsburgh, PA).

#### EEG recording and Neurofeedback

**Fig. 3:**
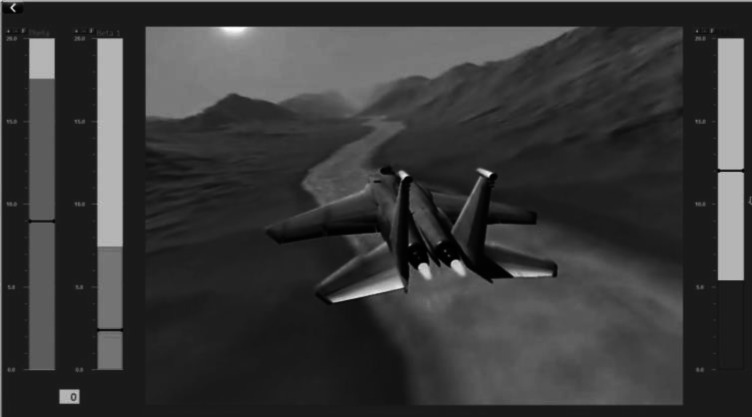
NFT feedback display; a video of an airplane flying over a mountainous terrain that only proceeds if the theta power (first bar from left) was below the pre-set threshold (horizontal black line on the bar) and the beta power (second bar from left) was above the pre-set threshold simultaneously. The bar on the right represents eye-blinks and muscle artifacts. This image is a screenshot of NFT feedback in BioTrace Software (Mind Media B.V., The Netherlands)

The TBR Neurofeedback signal was measured and applied by a NeXus-4 amplifier and recording system with BioTrace Software (Mind Media B.V., The Netherlands). The NeXus-4 amplifier is a DC amplifier in which EEG is sampled at 1024 Hz. One NeXus Ag/AgCl disposable electrode was applied on the participant’s scalp between locations Cz and FCz. A ground and a reference electrode were placed on the jaw and right-ear mastoid respectively. Additionally, nine extra in-cap electrodes (BioSemi, The Netherlands) were added on locations F3, Fz, F4, C3, Cz, C4, P3, Pz, P4, with one reference electrode on the left-ear mastoid, during every session. Data from these electrodes were collected with the Biosemi ActiveTwo DC amplifier. Both devices were active during all measurements, except during the sham-NFT sessions; then both the NeXus and the BioSemi system were attached but only the BioSemi system was active. Each session had a four-minute baseline measurement and a 25-min measurement-only, active-NFT or sham-NFT. Active and sham neurofeedback were provided by BioTrace Software. Per time window of 15 s, individualized thresholds were automatically reset in a way that, based on the previous 15 s, the feedback signal would likely indicate successful performance for ± 80% of the time, resulting in a standardized NFT protocol. Before feedback onset, measurement started 15 s earlier to determine the thresholds. When the EEG theta power went below the threshold, and the beta power above its threshold simultaneously, the participant was rewarded by the continuation of a video (a simulation of an airplane flying over a mountainous terrain, Fig. 3). If they failed to reach these thresholds, the video stopped. Theta and beta amplitudes were online filtered with a 4 Hz high-pass and 7 Hz low-pass filter for theta and a 13 Hz high-pass and 30 Hz low-pass filter for beta. Online calculation of theta and beta amplitude (= feedback resolution) was done in epochs of 125 ms using a moving time window; at every data sample (sampling rate was 256 per second) calculation was done over the last 125 ms of that sample. These amplitude values were calculated by taking the root mean square (RMS) of the band-pass filtered data. Since online Fast Fourier transformation needs at least 2 s to calculate amplitudes; RMS is a representative and practical method of online calculation (Nitschke et al. 1998). Feedback by video continuation therefore appeared continuously when theta and beta were below and above the threshold for 125 ms. The theta and beta amplitudes were visualized in separate bar graphs on the screen, next to the video. A third ‘inhibit’ bar represented eye blinks or muscle artifacts. The filter of this inhibit band was set at 2–3 Hz for eyeblinks and above 60 Hz for muscle artifacts. If the amplitude of the eyeblinks or muscle artifacts exceeded its threshold, no feedback was provided. The theta bar was a fluctuating bar in blue and beta a fluctuating bar in red. The participants were instructed which bar (beta) needed to go up above a threshold (small black stripe) and which bar (theta) needed to go down below a threshold, and in this way they had to keep the video running. No instructions about how to influence their EEG spectrum were given. With respect to this, the participants were only told to ‘sit still’ and ‘not to tense their face or jaws’ (to reduce interference from muscle activity and to prevent increased beta activity resulting from such volitional motoric action).

The sham-NFT was a previously recorded active-NFT session of a participant from Group A (received 14 active-NFT sessions). Every participant in Group C (sham-NFT) was matched to another participant in Group A and received the active-NFT video per session of their matched participant. That is, participants in Group C at session 4 saw the video and bars moving as if it was caused by their own EEG, however they actually watched the feedback that their matched participant from Group A in session 4 received. In this way, participants receiving sham-NFT were not able to influence the theta or beta bar graphs nor the continuation of the video. By matching every participant in Group C to a real participant in Group A, we kept the sham-NFT realistic for the participants in Group C, providing an accurate ‘yoked control’ procedure controlling for possible effects of motivation as a result of the received feedback.

### Procedure

#### General procedure

Testing took place at Leiden University, between February 2016 until May 2016. All participants visited the lab 17 times. In all sessions, participants received state questionnaires, a four-minute EEG passive baseline resting state measurement followed by either a measurement-only, an active NFT or a sham-NFT (all 25 min; for an overview see Fig. 2). Sessions were planned minimally three times a week with a maximum of five times a week. Every session took place on a separate day with a maximum of three days in between. One session approximately took between 60–70 min. The complete experiment therefore took 17–20 h per participant in approximately four weeks. At the end of every session, all participants performed a 10-min cognitive control task (Bishop 2009). We included this task to pilot its extensively repeated use in a multiple baseline design for future studies. Results on this task are irrelevant for the current hypotheses and therefore the task and its outcomes will not be further reported.

#### First session

During the first session, participants were asked to sign informed consent in which they were informed about the pay-off scheme regarding financial compensation. After signing informed consent, a questionnaire about general and medical information was completed including questions about drug use and health. Participants started with the STAI-T, ACS, BAS, STAI-S and SACS; in that order. Subsequently, preparation of the EEG equipment started and the participants continued with the four-minute passive baseline measurement followed by a 25 min ‘measurement-only’ part.

#### Session 2–16

The second session till the sixteenth session, all maintained the same procedure, except that the fourth session till the sixteenth session could either include a measurement-only, an active NFT plus EEG measurement or sham-NFT plus EEG measurement (see Fig. 2). Participants always started with completing the STAI-S and SACS. This was followed by the EEG four-minute passive baseline measurement and the 25-min measurement-only (session 2 and 3 for all groups and 2–9 for Group B), active-NFT (session 4–16 for Group A and session 10–16 for Group B) or sham-NFT (session 4–16 for Group C).

#### Last session (17)

The last session started with completion of the STAI-T, BAS and trait ACS questionnaires, followed again by the STAI-S and SACS questionnaires, EEG four-minute passive baseline measurement and the 25-min active NFT or sham-NFT. All participants ended with a funneled debriefing interview. In this interview the participants were asked how they experienced the study, what kind of mental methods they used to become successful in the training, and which experimental group they thought they were in and why. After completing the interview, participants received a financial reward for their participation.

### Data reduction and analysis

Data processing was done using Brain Vision Analyzer V2.0.4 (Brain Products GmbH, Germany). Data was high-pass filtered at 0.1 Hz, low-pass filtered at 100-Hz and a 50-Hz notch filter was applied. The data was automatically corrected for ocular artifacts (Gratton et al. 1983) in segments of 4 s. Remaining segments containing muscle movements, amplitudes above 200 µV or other artifacts were removed. For offline amplitude calculation, Fast Fourier transformation (Hamming window length 10%) was applied for theta (4–7 Hz) and beta (13–30 Hz) at C3, Cz, and C4 positions. Amplitude values were calculated by taking amplitude spectral density (µV*Hz). Amplitude squared provided the power values. Central theta and beta power was calculated by taking the average of C3, Cz and C4 positions, and central TBR in turn was calculated by dividing central theta power by central beta power. Central TBR was chosen as outcome variable of focus because the NeXus sensor for active-NFT was placed between Cz and FCz positions, however we have exploratively looked at frontal average TBR too, as well as theta, beta, beta 1 (13–20 Hz) and beta 2 (21–30 Hz) separately. All raw data are freely available on (https://doi.org/10.7910/DVN/FJD7TQ).

For interpreting the results, primarily visual inspection was used to determine the effectiveness of the active-NFT. If central TBR in Group A would reduce shortly after the introduction of the active-NFT (and after a comparable delay across the four participants) compared to no changes in Group C, the experiment would provide compelling evidence for the effectiveness of active-NFT. The effect would be even more strongly supported if a similar reduction was seen in Group A and B (after an equal number of active-NFT sessions, regardless the longer duration of the baseline). These changes after onset in Group A and Group B are assumed to be absent in Group C, where we expected no changes. The expected change in Group B could be considered as a direct replication of the effect in Group A. Furthermore, we expected differences in central TBR between Group A, B and C at the last session compared to the first session, with Group A showing the strongest reduction in central TBR (after performing the most active-NFT sessions), and Group C showing the weakest reduction in central TBR (no active NFT sessions). Primarily, we expected to see a consistent reduction in central TBR over active-NFT measurements, though we have inspected the four-minute passive baseline measurements as well, despite its smaller chance to detect any effect of active NFT. Also, besides inspecting changes in central TBR over all sessions, we have inspected changes in central TBR at the end of every session (average of last five minutes) and changes over time (25 min) within sessions too, since fluctuations might have occurred across the 25 min that could remain undetected when only inspecting session averages.

Trait anxiety and self-reported trait attentional control were measured at the start and the end of the study to exploratively relate these measures to possible changes in TBR as indication of potential unwanted side-effects. State anxiety and state-AC were assessed during every session to allow closer observation of such potential side effects.

## Results

### Participants

**Fig. 4:**
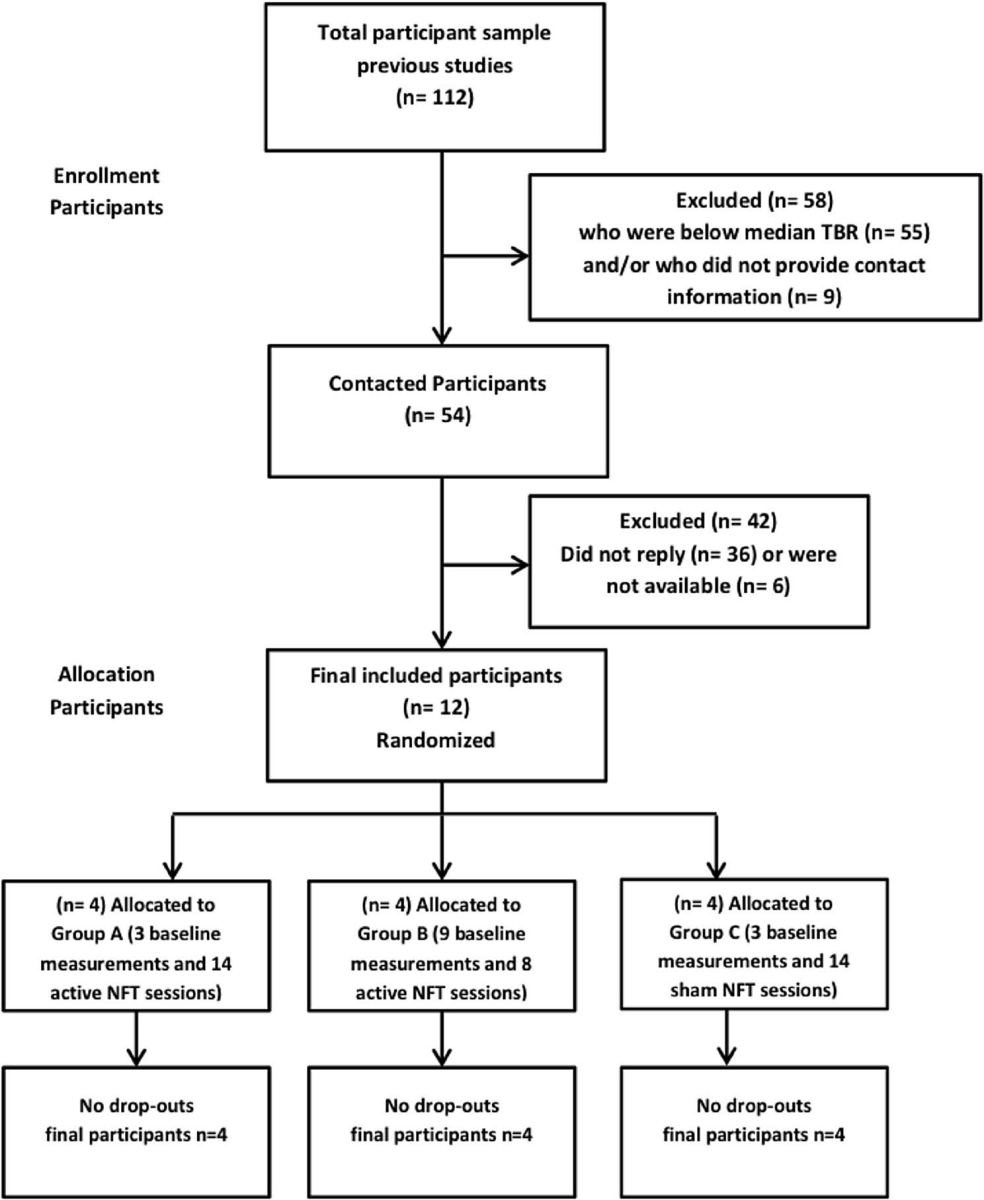
Flow diagram of participant recruitment and testing

Twelve participants were selected, and all completed the 17 sessions (for a flow diagram of participant selection, see Fig. 4). Age, baseline TBR and questionnaire scores per participant and per group during the first session are summarized in Table 1. The first baseline measurement of the selected 12 participants in the current study showed a frontal TBR of *M* = 1.51, *SD* = 0.76, median = 1.43). Although this was somewhat lower than their frontal TBR during their pre-selection measurement (*M* = 1.68, *SD* = 0.55, median = 1.47), it was still noticeably higher than the frontal TBR that was observed in the *N* = 56 unselected sample that the preselection was based on (*M* = 1.26, *SD* = 0.54, median = 1.13) and represented the 45th–88th percentile score of this unselected sample. In sum, also at the time of testing, the sample had elevated frontal TBR.

**Table 1:** Demographic information of all participants and means per group. Note TBR = frontal theta/beta ratio; M = mean, ± is standard deviation; Group A is early active NFT onset; Group B is late active NFT onset; Group C is sham NFT

	Age	Baseline TBR	ACS score	STAI trait score	BAS total score
Group A	*M* = 20.5 ± 1	*M* = 1.74 ± 0.81	*M* = 55.5 ± 5.97	*M* = 37 ± 2.16	*M* = 43 ± 4.54
Participant# 2	21	1.72	48	37	37
4	21	1.19	58	36	42
5	19	2.89	54	35	46
10	21	1.15	62	40	47
Group B	*M* = 21.25 ± 1.25	*M* = 1.80 ± 0.51	*M* = 50.5 ± 11	*M* = 45.25 ± 9.78	*M* = 38 ± 7.48
Participant# 1	21	2.50	45	36	36
7	21	1.36	45	56	38
8	20	1.88	45	51	30
11	23	1.48	67	38	48
Group C	*M* = 21.25 ± 0.96	*M* = 1.5 ± 0.32	*M* = 53.75 ± 7.89	*M* = 35 ± 4.08	*M* = 44.5 ± 3.42
Participant# 3	21	1.93	50	38	44
6	20	1.44	57	39	46
9	22	1.16	63	32	40
12	22	1.47	45	31	48
Total mean	*M* = 21 ± 1.04	*M* = 1.68 ± 0.55	*M* = 53.25 ± 8.02	*M* = 39.10 ± 7.31	*M* = 41.83 ± 5.70

### Passive baseline between sessions

**Fig. 5:**
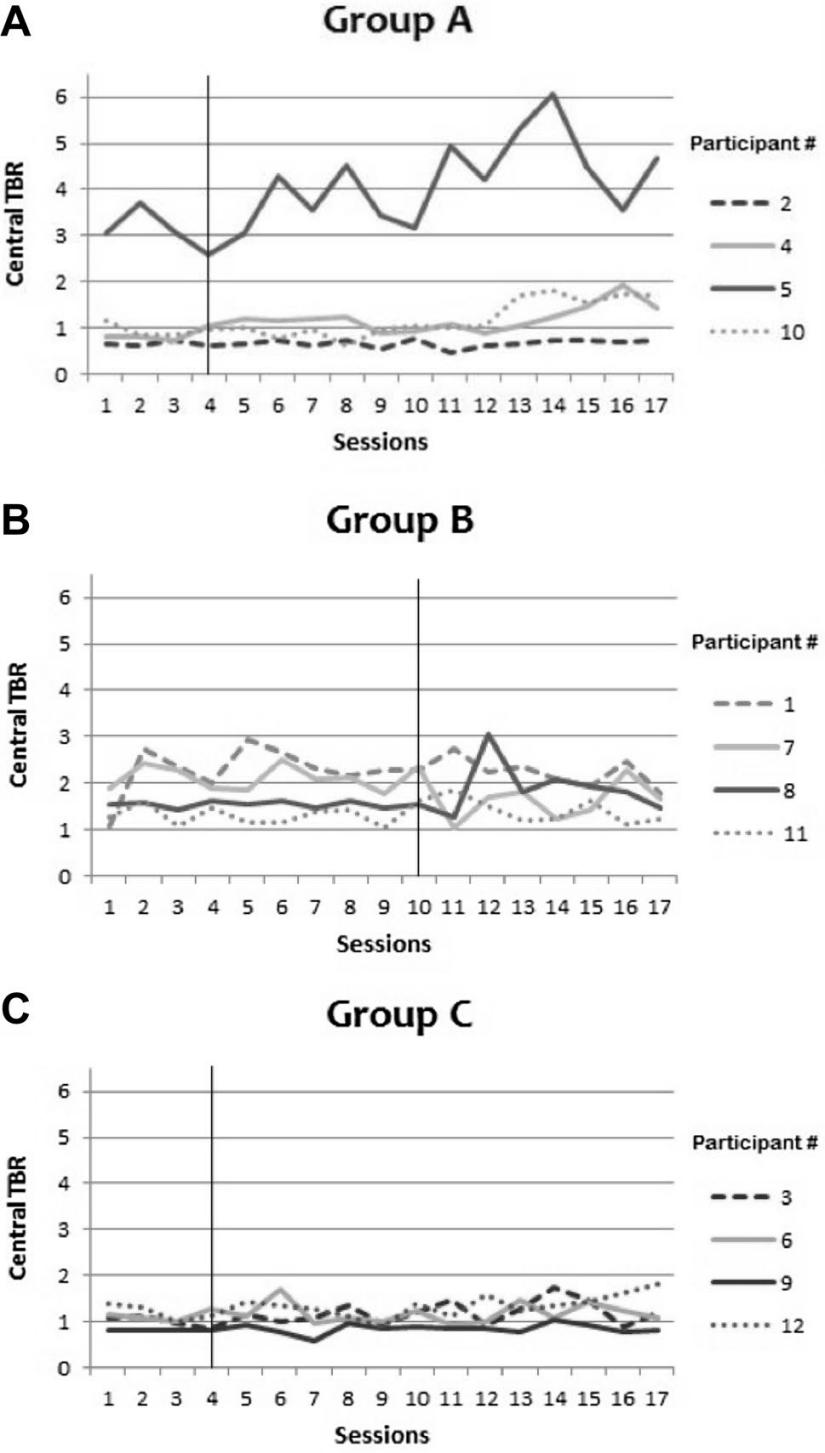
**a**–**c** Central TBR averages per 4 min baseline measurement per participant (lines) in Group A, B and C (Group A is early active NFT onset; Group B is late active NFT onset; Group C is sham NFT). The thin black vertical lines represent when the active or sham NFT started

Each session started with a four-minute passive baseline measurement. Figure 5 shows the pattern of the average central TBR on the four-minute passive baseline per participant. A vertical line indicates the start of active-NFT or sham-NFT. We hypothesized that central TBR would reduce after the onset of active-NFT (in Group A and Group B) but would not show a consistent decrease or increase after the onset of sham-NFT (in Group C). Visual inspection provided no support for such pattern. Passive baseline central TBR did not consistently change during the study, although some apparently random fluctuations between sessions were observed. This was invariably the case for all participants in both Groups A and B. No consistent increase or decrease of baseline central TBR was observed at any point in time in any of the participants. None of the participants that received sham-NFT showed a consistent decrease or increase after the onset of sham-NFT (Group C).

### Average TBR during the active training phase of the sessions

**Fig. 6:**
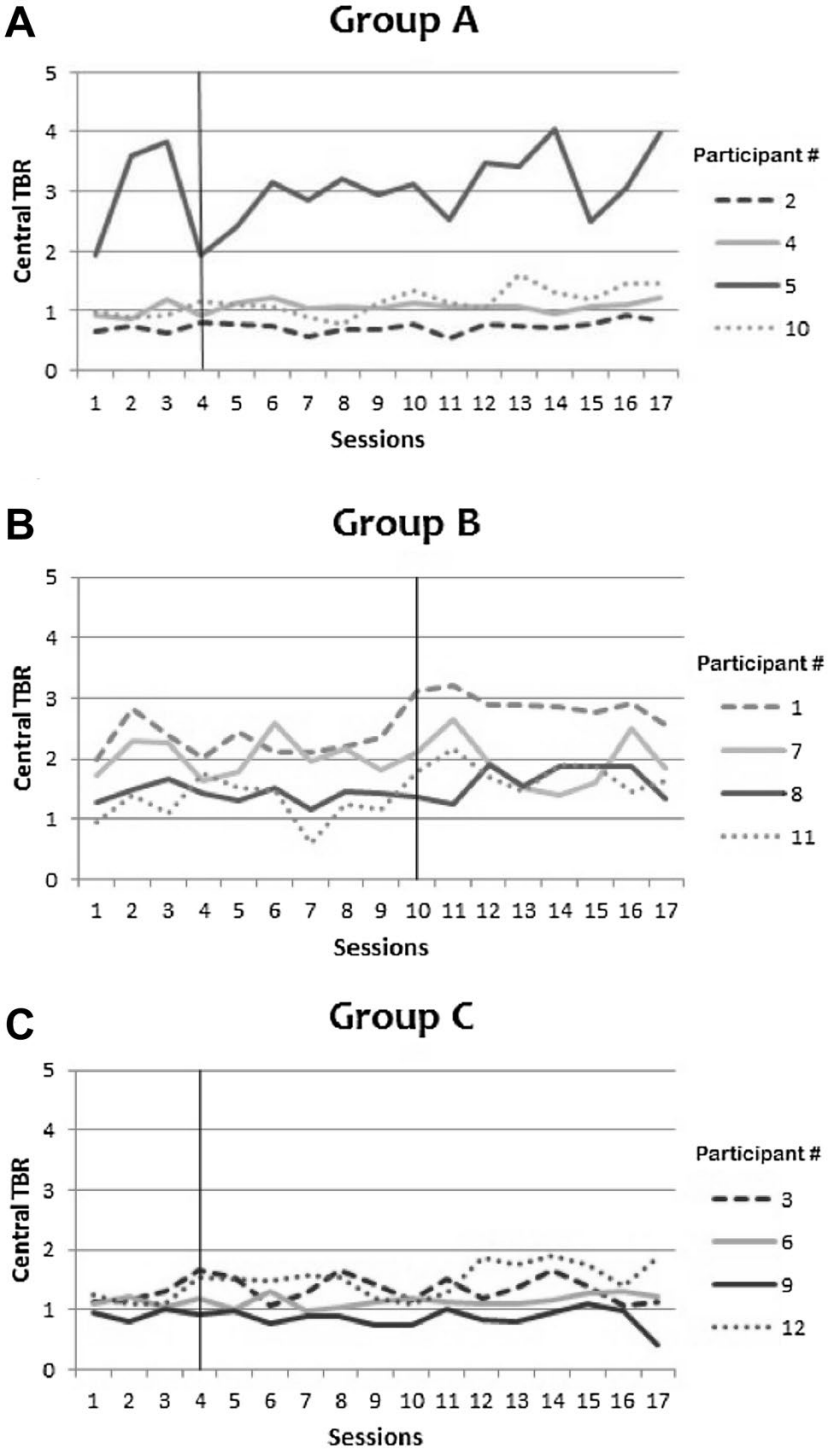
**a**-**c** Central TBR averages during the 25-min sessions of measurement only or active NFT or sham NFT training for Groups A, B and C (Group A is early active NFT onset; Group B is late active NFT onset; Group C is sham NFT)

Next, we inspected the pattern of average central TBR on the 25-min measurements (measurement-only, active-NFT or sham-NFT). This pattern was visually inspected per participant and by calculating the average central TBR per session per participant (Fig. 6). No consistent decrease was observed in any of the participants across sessions on central TBR after active-NFT onset and no differences between participants from different groups were apparent (nor for any other EEG parameter; see online data repository). In group B (delayed active NFT), participant # 1 seems to show a relatively stable *increase* in TBR, but this trend started before onset of active NFT which does not support an effect of active NFT. Additionally, no group differences were observed between Group A, B, and C in central TBR pattern over sessions.

### Last five minutes of training phase

**Fig. 7:**
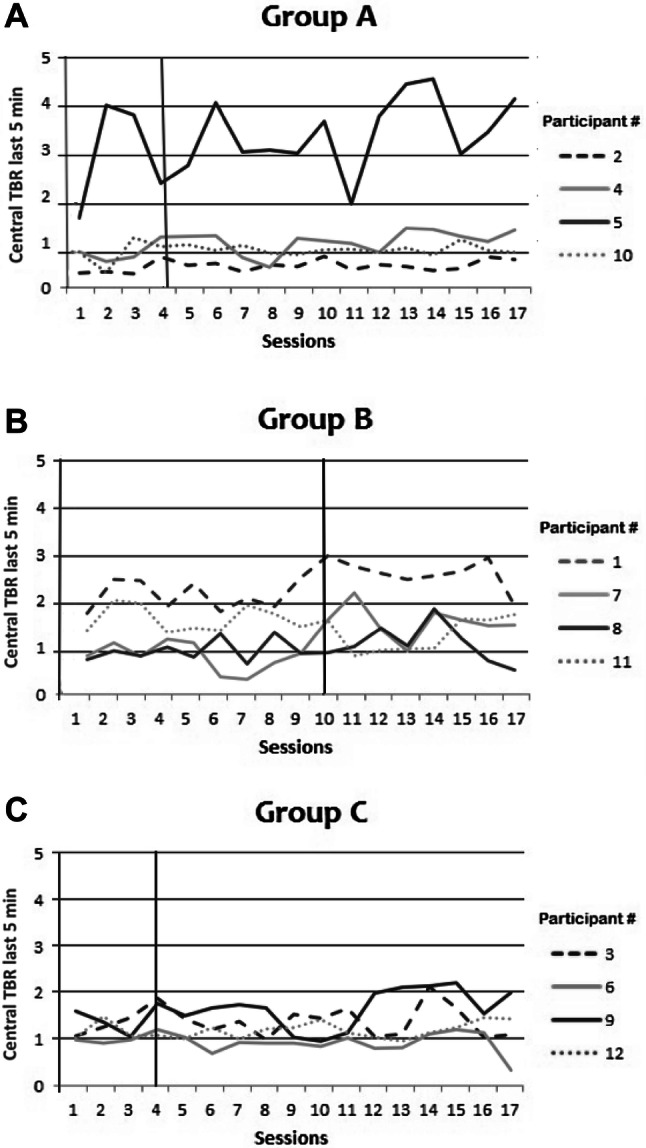
**a**–**c** Central TBR averages of last 5 min per measurement-only/active/sham NFT session per participant in Group A, B, or C (Group A is early active NFT onset; Group B is late active NFT onset; Group C is sham NFT)

The possibility exists that calculating an average over a longer period of time will obscure any delayed within-session effects of active-NFT. In other words, active-NFT might reduce central TBR at the end of sessions only, for example because the learning-process takes time. To check for this possibility, we explored changes between sessions in average central TBR during the last five minutes of every session. Figure 7a–c show the pattern of the central TBR over sessions per participant of the averaged final five minutes. In Groups A (early active NFT onset) and B (late active NFT onset), none of the participants show a reliable change for the last 5 min of the sessions after onset of active NFT. Also, no reliable change was observed in any participants in Group C (sham).

### Closer inspection per case

**Fig. 8:**
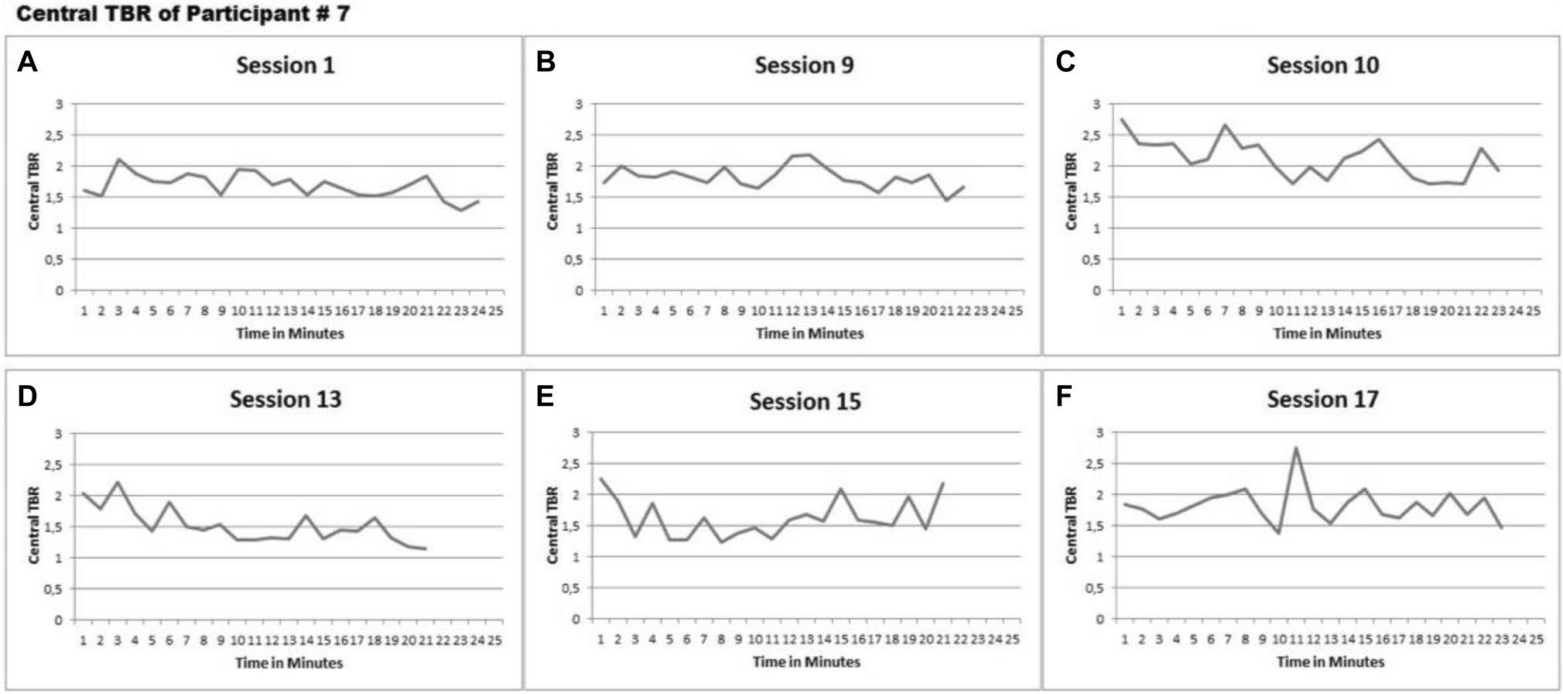
**a**–**f** Central TBR over time for participant 7 within; session 1 (**a**) session 9 as the last measurement-only session (**b**), session 10 as the first active-NFT session (**c**), session 13 and 15 as two in between active NFT sessions (**a**, **e**) and session 17 as the last session (**f**)

Finally, we examined whether any consistent change in central TBR could be detected *within* the sessions for any participant. We calculated the average central TBR per minute for every session and plotted these over time (25 min on the x-axis) for each session and participant. All plots can be created and viewed with our data available online (https://doi.org/10.7910/DVN/FJD7TQ). Developments of central TBR over time were examined per participant within sessions. The effect of active-NFT might for example have been driven by motivation, or inhibited by fatigue, factors that are likely a function of the duration of a session and that are different for each session and participant. Visual inspection did not reveal clear change for any of the participants over central TBR within the active-NFT sessions. We here present some detailed data as example for one of the ‘best cases’, showing some kind of change in central TBR/active-NFT effect. For two out of four participants (participant # 7 and 11) in Group B, central TBR seemed to decrease within some active-NFT sessions compared to the measurement-only sessions. Here, we present these detailed data only for participant 7 who seemed to maybe show the most change (Fig. 8c, d. See https://doi.org/10.7910/DVN/FJD7TQ for all data of all participants). The reduction in central TBR occurred in active-NFT sessions 10 and 13 but not anymore in sessions 15 or 17. Furthermore, central TBR always started at approximately the same value in all active NFT sessions of participant 7. The other participant (#11) with possible change in some session, similarly showed no retention of the slight change during subsequent sessions. The data therefore do not show any transfer of a learning process caused by the active-NFT intervention.

### State anxiety and state AC over time

**Fig. 9:**
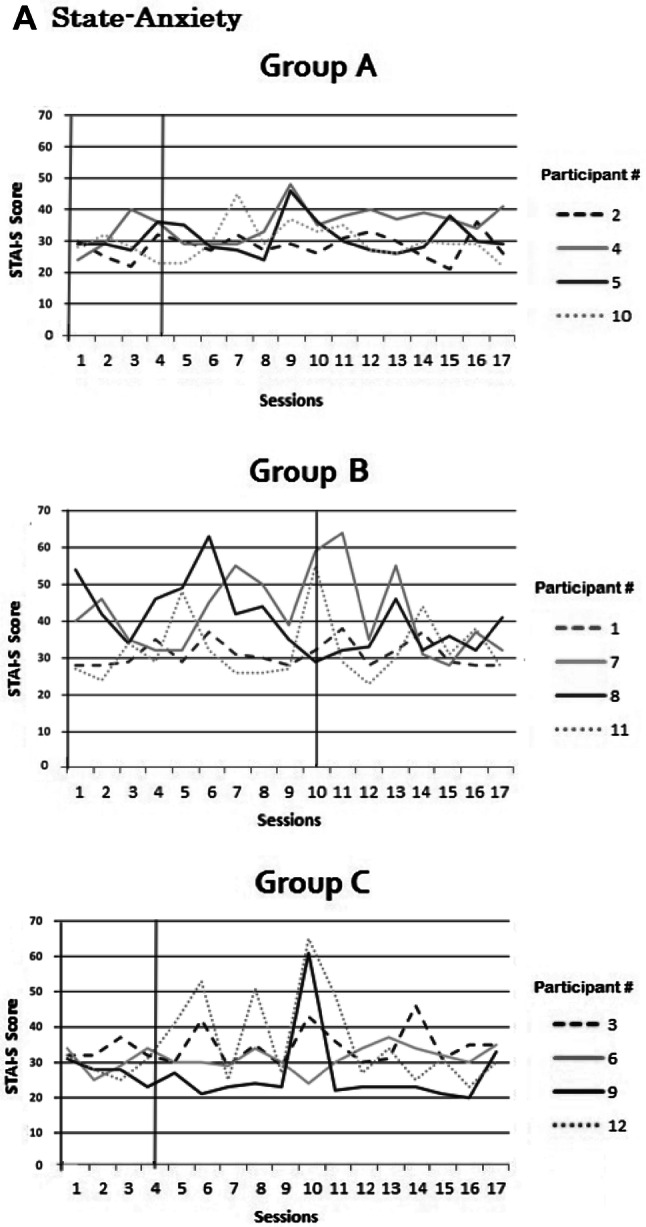

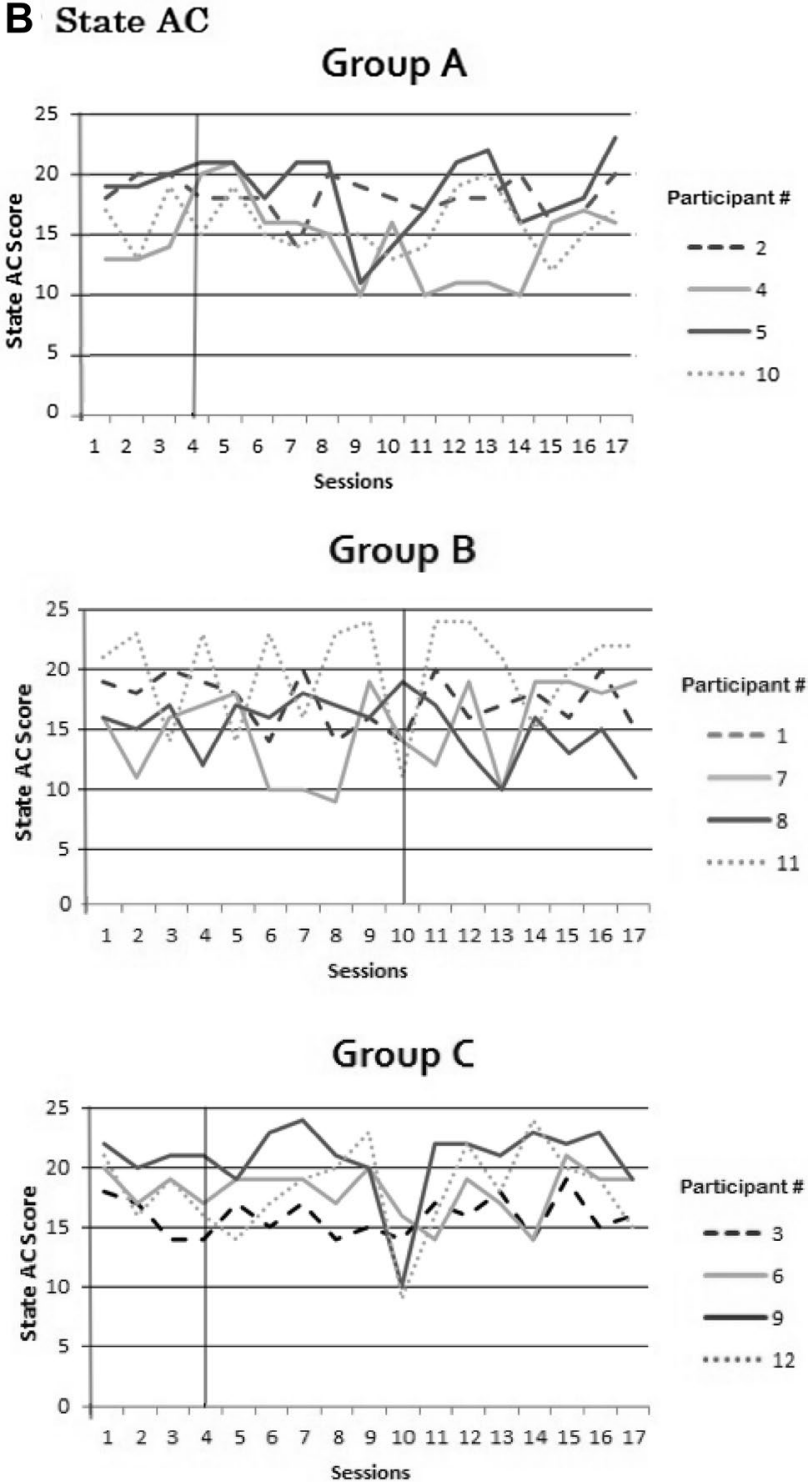
**a**, **b** Scores on state anxiety (**a**) and state attentional control (**b**) per session per participant in Group A, B or C (Group A is early active NFT onset; Group B is late active NFT onset; Group C is sham NFT)

To check for potential adverse effects, scores on STAI-S and SACS were plotted for all participants over all sessions and visually inspected for changes over time. The plots show that for all participants in all three groups, STAI-S and SACS did not show any consistent increase or decrease over sessions (Fig. 9a, b). Active-NFT sessions therefore did not seem to induce any adverse effects on state anxiety or state attentional control. Finally, scores on trait anxiety, ACS and BAS scores did not show any changes as measured on the first session compared to the last session. No reliable adverse effects were observed nor reported by any of the participants.

### Motivation and debriefing

All participants generally reported to be motivated performing the NFT over all sessions (*M* = 3.35; range 1–4 with 4 being most motivated), although for all three Groups (A, B and C) there was a small drop in motivation between the 10th and the 12th session. Motivation returned to their initial level after the 12th session. All participants received a funneled debriefing interview after the final session of the study, and it became clear that two out of the four participants that received sham-NFT (Group C) were not sure whether they were in the sham-controlled group (60% chance of being in the sham controlled group) whilst the two other participants in Group C thought that they had received an active-NFT (70% chance).

## Discussion

In this study we aimed to reduce central TBR with NFT in healthy individuals with elevated TBR. The results indicate that active-NFT did not alter TBR in any way. No consistent within-session change of TBR was found on either the passive baseline measurement or 25-min active training measurement nor was there any evidence of between-session change. This suggests that the active-NFT did not induce any changes in EEG measurements of interest. State anxiety and state attentional control did not show a consistent change after active-NFT onset either. All participants reported to remain motivated performing the active- or sham-NFT however, and participants that received sham-NFT indicated that they believed to have received an active-NFT.

The present study was a first step towards intervention studies in a healthy population with elevated TBR. We expected that NFT would reduce TBR in healthy participants. Changes in EEG were the primary outcome, as these changes are likely easier to detect and more consistent than changes at the more multifaceted and complex behavioral level. We used a multiple baseline case series design for a detailed study of all NFT effects.

Our finding that active-NFT did not induce any consistent reduction or increase in TBR in healthy individuals is in line with the results of Doppelmayr and Weber (2011) who performed a randomized controlled trial in 14 healthy participants. Thirty active-NFT sessions did not induce changes in EEG TBR or the separate theta or beta frequency bands. Their results do not provide explanation why the active-NFT did not alter TBR. Possibly, some changes were simply not detected because TBR changes were not inspected within-sessions. By using a multiple baseline case series design, we provide a detailed view of what precisely happened with EEG TBR over time after the onset of active-NFT over sessions, as well as a precise view of TBR changes within active NFT sessions, over the course of 25 min. None of our detailed observations provided any evidence that active-NFT had an effect on the primary outcome variable, the EEG spectrum level of TBR, replicating the results of Doppelmayr and Weber (2011).

In particular, we had the ability to visually inspect what exactly happened with TBR over time on different levels of the data, between all participants and all conditions. First of all, we inspected the passive baseline measurement, which was done in four minutes before every 25-min active measurement. No consistent decrease or increase of TBR was found in any of the participants. Yet, the passive baseline measurement was no main outcome variable because a longitudinal change in TBR was found to be more difficult to achieve than a direct change in TBR (van Doren et al. 2017). The main outcome measure was average TBR over time per session, for which we expected a consistent decrease in central TBR with a comparable lag after the first active-NFT session for the two active NFT groups. No such decrease or any other consistent change in central TBR was observed, making it unlikely that with our NFT procedures, TBR can be reduced in healthy participants. If any NFT induced changes would not transfer between sessions and take a long time within-sessions to occur, then reduced TBR might have been only visible at the end of the session, but no consistent change in central TBR was observed in any of the participants in the last-five-minute averages either. Finally, it might have been possible that non-linear fluctuations in TBR occurred over the 25-min active measurement, which would remain undetected when solely inspecting session averages. Only two participants in Group B showed the least bit of evidence of consistent reduction in TBR over time within the first few active NFT sessions. It became clear however that in their fifth active-NFT session this apparent TBR reduction was no longer discernable and again from this session onwards no consistent change in TBR was observed. Detailed analyses of data per case therefore did not show a transfer of learning caused by the active-NFT intervention in any way.

State anxiety and state attentional control were included for prudent use of an intervention like active-NFT of which no details on its side effects in a healthy population are known yet. The aim was to check carefully if state anxiety did not increase and state attentional control did not decrease. Plots of STAI-S and SACS scores for all participants over all sessions were visually inspected on changes over time and no consistent change in either state anxiety or state attentional control was observed. We advise future studies to monitor unwanted effects on anxiety, attentional control and hedonically motivated behavior, as existing literature provides some reasons for concern (Angelidis et al. 2016; Massar et al. 2012, 2014; Putman et al. 2010). Similar arguments remain for the question if TBR down-training might actually reduce working memory in healthy participants (see Enriquez-Geppert et al. 2014). Regarding the main research purposes of our study, the data does not provide any evidence for active-NFT causing changes to the EEG spectrum. We were purely interested in reducing TBR and to assess whether the NFT manipulation can be considered successful in doing this. Janssen and colleagues aimed to down-train TBR with NFT in children diagnosed with ADHD, and found no effects of NFT after 30 sessions on theta, however they found a significant increase in beta over sessions (Janssen et al. 2016). These authors noted that this increase in beta activity was possibly a result of volitional motoric action as some participants reported to occasionally apply this during the active-NFT and cortical beta power is associated with motor control (Hammond et al. 2001). In a more recent study, the same research group moreover compared long term effects of TBR NFT to methylphenidate and a semi-active control intervention on EEG power spectra, in children with ADHD. No differences were found in EEG measures between experimental groups, suggesting that TBR NFT does not have long term effects in children with ADHD (Janssen et al. 2020).

When debriefing our participants, almost all indicated having used a different ‘technique’ to reduce the theta and increase the beta band, ranging from counting to imagining music. Neurofeedback is generally seen as an operant conditioning process (Kamiya 2011; Strehl 2014). However, other aspects like skill learning ability and motivation turned out to have a strong influence too (Roberts et al. 1989; Hofmann et al. 2012; Strehl 2014). There seems to be a strong impact of feedback reinforcement, application of trial and error and transferring learned skills into everyday life (Abikoff 2009; Mazur 2002) making any effect of NFT dependent on individual differences. Also, it should be taken into account that the single-blind nature of this study might involuntarily have affected the interaction between the experimenter and participant. However, the instructions that the experimenters provided were standardized and no signs of such experimenter effects were reported by the participants in the debriefing. Moreover, it should be mentioned that our sample is not generalizable to the entire population in terms of age and gender, as we have measured female university students between 19 and 24 years old with elevated TBR (with respect to their previous study samples) only.

A few methodological choices in our study must be highlighted here, in order to best interpret the data and to increase the informative value of this report’s null findings. Firstly, the use of automatic threshold regulation might not correspond to the prerequisites of shaping a learning process (Gruzelier 2014b). It is reasoned that not all individuals learn at the same speed, and the above-mentioned individual differences may also play a role. Manually adjusted thresholds, usually by a trained clinician, is suggested as a solution to this potential problem (Bazanova et al. 2007; Bazanova and Vernon 2014; Klimesh 1999) but has obvious experimental-methodological drawbacks. However, positive findings for successful regulation of beta or theta activity have been reported for other studies that did not use manual threshold adjustment. For instance, Lubar et al. (1995) used an automatic threshold scheme. Leins et al. (2007) used a reward method that automatically changed every 15 sessions. Fuchs et al, (2003) also used automatic thresholds when applying SMR/beta ratio neurofeedback in children diagnosed with ADHD. Neurofeedback training significantly reduced ADHD related behavioral problems. In their study, the thresholds were set to accept the signal 70% of the time, which is similar to the protocol as used in the current study. Since these studies did report changes in EEG activity, it seems unlikely that our null findings result from our use of that method. In general, no studies have been conducted that directly compared automatic thresholding to manual thresholding when using NFT, making it difficult to draw firm conclusions on this issue that go beyond simple observation (Gruzelier 2014b).

Secondly, our protocol used a ‘continuous’ video feedback procedure. It has been argued that entertaining feedback might strengthen reinforcement associated with the stimulus rather than a specific brain-behavior response, suggesting that discrete feedback (e.g., earning points) might be more effective on the long-term (Egner and Sterman 2006). However, Butnik (2005) described cases in which children diagnosed with ADHD successfully reduce or increase the targeted frequency bands when being submitted to video-neurofeedback trainings. Furthermore, Kouijzer et al. (2009) successfully reduced excessive theta power when applying video feedback in children with autism spectrum disorders. No studies have compared the effectiveness of continuous vs. discrete feedback.

Thirdly, the number of sessions used in NFT varies widely in the literature, and is usually dependent on the trained population as well as the specific protocol that is used (for a review see Enriquez-Geppert et al. 2017). Reiner et al. (2014) found posterior theta to change already after one session, followed by some studies that observed clear changes in alpha after only one neurofeedback training (Escolano et al. 2014; Ros et al. 2014; Xiong et al. 2014). Also, Enriquez-Geppert et al. (2014) found frontal-midline theta to change after eight sessions of NFT, making it difficult to explain the absence of changes in theta after 14 training sessions in the current study. The duration of a single session is usually between 20 and 40 min, dependent on the participant’s ability to focus on the training, which varies across health status and age (see Enriquez-Geppert et al. 2017; Gruzelier 2014b for systematic reviews). For these reasons and because of the complete lack of EEG change throughout the entire duration of our study, it seems unlikely that we would have observed effects after more sessions.

Fourthly, we opted to select participants with elevated TBR scores, because such participants might respond better to the training. Although mean TBR had decreased somewhat between the pre-selection measurement and the start of the current study some six months later (regression to the mean may have occurred), their TBR was still clearly above the TBR as observed in the unselected samples. For potential application of TBR NFT to increase cognitive performance, we had chosen to study the effectiveness of TBR in individuals with a mildly elevated TBR, also because it is not unlikely (though undocumented) that such participants might respond better to the training. Nevertheless, future studies might refrain from such a pre-selection, which can possibly contribute to the generalizability of study outcomes.

In summary, we found no evidence that TBR-targeted NFT affects TBR in healthy participants. Although it is possible that different NFT protocols may lead to different results, the present findings indicate that TBR is not affected by NFT, as implemented in the current study (using automatic thresholds, video feedback with a maximum of 14 sessions). The current study had several methodically strong features; a case series multiple baseline design that allowed us to inspect all EEG data per participant per session in detail, and control for unknown side effects. A sham controlled NFT group was included that, according to the funneled debriefing, let the participants believe that they received an active-NFT. We cannot identify convincing procedural limitations of our study that might serve to adequately explain the lack of positive result and thus consider these results a valid null-finding. These results are relevant given recent publications on TBR and its relation to executive cognitive control and affect regulation (Angelidis et al. 2016; Angelidis et al. 2018; Massar et al. 2014; Schutter and Honk 2005; Tortella-Feliu et al. 2014; van Son et al. 2018; Wischnewski et al. 2016) in healthy adults. The present results, which replicate and extend previous results by Doppelmayr and Weber (2011), do not suggest that TBR-targeted NFT will likely provide a tool to study causality of the relations between cognitive control and affect regulation. Furthermore, TBR NFT does not seem to be a promising candidate for human performance enhancement in these functional areas.
